# Supplementary material for: Hemp Flour Particle Size Affects the Quality and Nutritional Profile of the Enriched Functional Pasta
Source: Foods. 2023 Feb 10;12(4):774. doi: 10.3390/foods12040774 (PMC9955999; doi:10.3390/foods12040774)
Supplement: Supplementary file 1 [file foods-12-00774-s001.zip › foods-2176123-supplementary.pdf]

Hemp Flour Particle Size Affects the Quality and Nutritional Profile of the Enriched Functional Pasta

Sonia Bonacci [1], Vita Di Stefano [2,*], Fabiola Sciacca [3], Carla Buzzanca [2], Nino Virzì [3], Sergio Argento [4] and Maria Grazia Melilli [4]

[1] Department of Health Sciences, University Magna Græcia of Catanzaro, 88100 Catanzaro, Italy
[2] Department of Biological, Chemical and Pharmaceutical Sciences and Technologies, University of Palermo, 90133 Palermo, Italy
[3] CREA-Council for Agricultural Research and Economics-Research Centre for Cereal and Industrial Crops of Acireale, 00187 Roma, Italy
[4] National Council of Research, Institute of BioEconomy (CNR-IBE), 95126 Catania, Italy
* Correspondence: vita.distefano@unipa.it; Tel.: +39-09123891948

Table S1. Retention time (min.), coefficient of determination (R^2) and linear regression model of external standards used for phenolic compounds calibration.

Phenolic compound	(RT min)	R^2	Linear Regression
Chlorogenic acid	9.5	0.949	y=45.238x+36.969
Catechin	10.06	0.993	y=43,952x-12.043
Sinapic acid	11.57	0.995	y=36,497x+55.086
Hydroxycinnamic acid	12.09	0.986	y=46.141x+55.383
Benzoic acid	13.27	0.993	y=54.045x-72.121
Kaempferol	14.97	0.984	y=48.708x+116.66
Rutin	16.2	0.983	y=48.917x+102.65

Table S2. Retention time (min.), coefficient of determination (R^2) and linear regression model of external standards used for aminoacids calibration.

Aminoacid	(RT min)	R^2	Linear Regression
L-Alanine	47.55	0.958	y=941627x-83.308
L-Arginine	34.57	0.936	y=4*(10^6)x-314.65
L-Aspartic Acid,	43.16	0.939	y=636759x+44.438
L-Cystine	55.06	0.988	y=1*(10^6)x-59.647
L-Glutamic Acid	43.32	0.949	y=541591x+41.209
L-Glycine	45.14	0.971	y=1*(10^6)x-61.835
L-Histidine	55.45	0.960	y=1*(10^6)x-160.23
L-Isoleucine	52.64	0.935	y=1*(10^6)x+77.756
L-Leucine	48.97	0.976	y=1*(10^6)x-61.269
L-Lysine	55.24	0.988	y=1*(10^6)x-59.647
L-Methionine	51.13	0.961	y=993983x-10.114
L-Phenylalanine	52.51	0.952	y=1*(10^6)x+15.772
L-Proline	56.80	0.939	y=285823x+31.393
L-Serine	42.22	0.940	y=1*(10^6)x+48.224
L-Threonine	44.16	0.955	y=925910x+57.567
L-Tyrosine	47.28	0.948	y=815696x+87.85
L-Valine	51.46	0.934	y=870016x+38.288
L-Tryptophan	56.55	0.976	y=3*(10^6)x+161.81
L-Asparagine	41.30	0.955	y=4*(10^6)x-313.23
L-Glutamine	41.20	0.951	y=4*(10^6)x-314.65

Table S3. Retention time (min.), formula, MS spectra, and accurate mass measurement of phenolic compounds identified

Phenolic compound	Formula	m/z [M-H]- calculated	m/z [M-H]- experimental	RT (min)
p-Hydroxybenzoic acid	$C_7H_6O_3$	137.0239	137.0236	9.2
Protocatechuic acid	$C_7H_6O_4$	153.0188	153.0182	7.3
Hydroxycinnamic acid	$C_9H_8O_3$	163.0395	163.0395	11.4
Vanillic acid	$C_8H_8O_4$	167.0344	167.034	11.0
Caffeic acid	$C_9H_8O_4$	179.0344	179.0344	10.1
Ferulic acid	$C_{10}H_{10}O_4$	193.0501	193.0509	12.1
Sinapic acid	$C_{11}H_{12}O_5$	223.0606	223.0602	11.6
Catechin	$C_{15}H_{14}O_6$	289.0712	289.0699	8.9
N-trans-Caffeoyltyramine	$C_{17}H_{17}NO_4$	298.1079	298.1081	18.6
Chlorogenic acid	$C_{16}H_{18}O_9$	353.0873	353.0871	9.6
Cannabisin B	$C_{34}H_{32}N_2O_8$	595.2080	595.2078	12.3
Cannaflavin C	$C_{26}H_{28}O_6$	609.2237	609.2240	12.8
